# Supplementary material for: A Randomized, Double-Blind, Placebo-Controlled Clinical Trial Assessing the Effects of Angelica Gigas Nakai Extract on Blood Triglycerides
Source: Nutrients. 2020 Jan 31;12(2):377. doi: 10.3390/nu12020377 (PMC7071255; doi:10.3390/nu12020377)
Supplement: Supplementary file 1 [file nutrients-12-00377-s001.pdf]

Supplemental files list

Supplementary Table S1. Nutritive components of the standard meal

**Standard meal menu**	**Weight (g)**	**Nutritive component**	**Content**
Steamed rice	210	Energy (Kcal)	640
Beef bulgogi (Korean beef stew)	100	Carbohydrates (g)	97.3
Soup	300	Lipids (g)	15.4
Stir-fried Vegetables(with mushroom)	100	Protein (g)	28.2
Radish kimchi	50	Fiber (g)	8.1

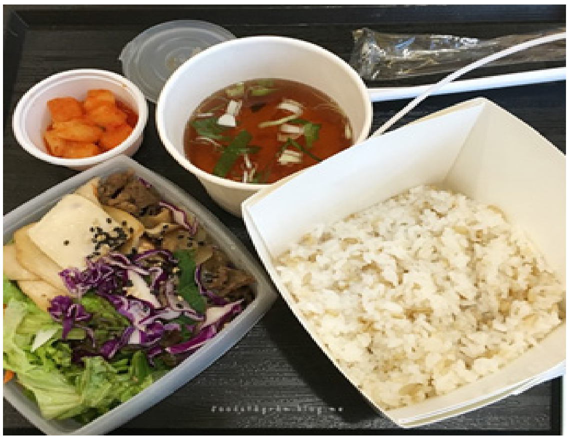

Supplementary Table S2. Laboratory profiles of the subjects in this study

Laboratory profiles (standard range)	AGNE group(n=48)			Placebo group (n=45)			
	Baseline	Week 12	p-value 1)	Baseline	Week 12	p-value 1)	p value2)
WBC (4.8–10.8×10³/μL)	5.8±1.4	5.9±1.7	0.309	5.7±1.5	5.7±1.3	0.717	0.289
RBC (4.2–5.4×100³/μL)	4.7±0.5	4.7±0.5	0.356	4.7±0.4	4.6±0.4	0.891	0.560
Hemoglobin (12–16g/dL)	14.2±1.5	14.1±1.5	0.244	14.2±1.5	14.1±1.4	0.618	0.636
Hematocrit (37–47%)	42.6±3.9	42.2±4.2	0.054	42.2±3.7	42.0±3.5	0.386	0.438
Platelet (130–450×10³/μL)	274.6±44.8	272.7±47.2	0.483	267.1±61.8	265.1±61.0	0.587	0.979
ALP (45–129IU/L)	67.3±20.0	67.6±20.7	0.811	66.8±19.9	68.0±18.3	0.305	0.566
GGT (8–48IU/L)	32.3±27.6	36.0±35.1	0.358	28.2±22.2	28.9±24.3	0.679	0.475
AST (12–33IU/L)	23.2±7.2	24.5±7.8	0.223	25.6±6.9	24.5±8.1	0.254	0.094
ALT (5–35IU/L)	29.7±17.2	32.6±21.4	0.160	30.4±15.3	27.7±16.8	0.081	0.029
Total bilirubin (0.2–1.2mg/dL)	0.96±0.4	0.95±0.4	0.927	0.87±0.3	0.84±0.4	0.529	0.647
Total protein (6.7–8.3g/dL)	7.5±0.4	7.5±0.3	0.621	7.4±0.4	7.4±0.4	0.960	0.725
Albumin (3.5–5.3g/dL)	4.4±0.2	4.5±0.2	0.059	4.4±0.2	4.4±0.2	0.513	0.257
BUN (8–23mg/dL)	14.3±2.9	14.9±3.4	0.067	15.1±3.8	15.0±3.9	0.705	0.169
Creatinine (0.7–1.7mg/dL)	0.68±0.2	0.7±0.1	0.898	0.69±0.2	0.7±0.2	0.285	0.439
Glucose (74–106mg/dL)	87.1±10.0	90.2±10.2	0.006	86.6±10.2	87.8±9.0	0.281	0.222
CK (50~200 IU/L)	102.3±48.0	111.7±84.5	0.266	101.8±50.2	122.1±169.0	0.383	0.659
LDH (218~472 IU/L)	365.8±46.8	372.5±51.9	0.304	375.0±62.5	359.3±51.6	0.010	0.012
pH (4.5~9.0)	6.5±0.9	6.3±0.7	0.058	6.4±0.8	6.3±0.7	0.464	0.355

Values are presented as mean ± SD
1) Analyzed by paired *t* test
2) Analyzed by Linear Mixed Model between groups
Abbreviations: WBC,White Blood Cell; RBC, Red Blood Cell; ALP; Alkaline Phosphatase; GGT, Gamma Glutamyl Transferase; AST;Aspartate Transaminase; ALT,Alanine Transaminase; BUN, Blood Urea Nitrogen; CK, creatine kinase;

LDH, lactate dehydrogenase.
